# Association between hemoglobin-to-red blood cell distribution width ratio and cognitive function in older US adults: a cross-sectional study based on the NHANES 2011–2014

**DOI:** 10.3389/fnagi.2025.1571159

**Published:** 2025-04-14

**Authors:** Fan Wang, Xiangyang Wang, Chaowei Wang, Hao Liu, Zhixiu Xu, Dongli Li, Xiaowen Zhao, Jialu Zhao, Shaomin Li, Jianhua Zhao

**Affiliations:** ^1^Henan Joint International Research Laboratory of Neurorestoratology for Senile Dementia, Xinxiang, China; ^2^Department of Neurology, The First Affiliated Hospital of Xinxiang Medical University, Xinxiang, China; ^3^Department of Neurosurgery, First Affiliated Hospital of Xinxiang Medical University, Xinxiang, China; ^4^Fujian Medical University, Fuzhou, China; ^5^Xinxiang Medical University, Xinxiang, China; ^6^Ann Romney Center for Neurologic Diseases, Brigham and Women’s Hospital, Harvard Medical School, Boston, MA, United States; ^7^Henan Joint International Research Laboratory of Neurorestoratology for Senile Dementia, Xinxiang, China; ^8^Henan Key Laboratory of Neurorestoratology and Protein Modification, Xinxiang, China

**Keywords:** hemoglobin, red cell distribution width, hemoglobin-to-red blood cell distribution width ratio, cognitive function, NHANES, including age, gender, genetics

## Abstract

**Introduction:**

The hemoglobin-to-red blood cell distribution width ratio (HRR) is acknowledged as a promising new prognostic biomarker. Nevertheless, investigations into its connection with cognitive function have yielded unclear results.

**Aim:**

This study aimed to investigate the association between the hemoglobin-to-red blood cell distribution width ratio (HRR) and cognitive function in older adults in the United States.

**Methods:**

This study utilized data obtained from the NHANES database, encompassing the years 2011 to 2014. The participant cohort consisted of elderly individuals aged 60 years and older, all of whom underwent thorough assessments of cognitive function, hemoglobin levels, and red blood cell width. We employed weighted logistic regression analysis, along with restricted cubic spline (RCS) curves and subgroup analyses, to rigorously evaluate the association between hemoglobin-to-red blood cell distribution width ratio (HRR) and cognitive function.

**Results:**

This study encompassed a total of 2,520 participants, with a mean age of 69.44 ± 6.76 years. After adjusting for multiple covariates, logistic regression analysis indicated a significant linear cognitive impairment between HRR and cognitive function. Specifically, each one-unit increase in HRR was associated with an 82% reduction in the probability of cognitive impairment among participants (OR = 0.18, 95% CI: 0.04–0.78). This relationship remained exist after HRR was categorized into tertiles. Participants in the highest HRR tertile exhibited a 42% lower likelihood of cognitive impairment compared to those in the lowest tertile (OR = 0.58; 95% CI: 0.37–0.91, *p* = 0.022). To validate the robustness of our findings, we conducted subgroup analyses, which consistently demonstrated stable results across all evaluated groups.

**Conclusion:**

This cross-sectional study revealed a significant negative correlation between HRR and cognitive function.

## Introduction

The prevalence and incidence of cognitive dysfunction are influenced by multiple factors. As age increases, the prevalence of cognitive dysfunction significantly rises, especially among the elderly population (Collyer et al., 2022). Additionally, individuals with a family history of the condition have a relatively higher risk of developing it. With the intensification of population aging, the prevalence of cognitive dysfunction is expected to continue to rise, and it is projected that the prevalence of dementia will reach 150 million cases by 2050 (GBD 2019 Dementia Forecasting Collaborators, 2022; Livingston et al., 2020). This will impose a heavy burden on society and families. Therefore, there is an urgent need to identify biological markers related to cognitive function.

Hemoglobin main function is to carry oxygen and transport it to all tissues and organs throughout the body. The concentration of hemoglobin is often used as an important indicator to assess whether an individual is anemic (Ahmed et al., 2020). In anemia, the number of red blood cells in the blood decreases, and the hemoglobin content drops, leading to hypoxia in the brain and potentially causing a series of health problems, including cognitive dysfunction (Winchester et al., 2018). RDW reflects the variability in the size of red blood cells. Elevated RDW has been linked to chronic inflammation, oxidative stress, cardiovascular diseases and Cognitive Impairment (Beydoun et al., 2020; Eyiol and Ertekin, 2024), all of which can adversely affect cognitive health. The hemoglobin to red cell distribution width ratio (HRR) is calculated by dividing hemoglobin (Hb) by red cell distribution width (RDW), and it is a new inflammatory marker proposed by Sun et al. (2016). Current research has found that this indicator is associated with various diseases such as stroke, depression, and chronic kidney disease, but its relationship with cognitive dysfunction remains unclear. This study aims to investigate the relationship between HRR and cognitive dysfunction.

## Methods

### Study population

This study utilized data from the National Health and Nutrition Examination Survey (NHANES), which was conducted by the National Center for Health Statistics (NCHS) and received approval from the NCHS Institutional Review Board (NCHS ERB Protocol Number: NHANES 2011–2012, Protocol #2011–2017 and NHANES 2013–2014, Continuation of Protocol #2011–2017), all registered participants provided written informed consent by signing the respective forms. NHANES employs a complex, multistage probability sampling design to ensure that the data accurately represent the non-institutionalized civilian population of the United States. Informed consent was obtained from all eligible participants before data collection commenced. All data are available on the official NHANES website^1^. Participants completed standardized home interviews and underwent health assessments at mobile examination centers to evaluate their medical and physiological conditions. Additionally, laboratory tests were performed to gather supplementary laboratory data.

**Figure 1:**
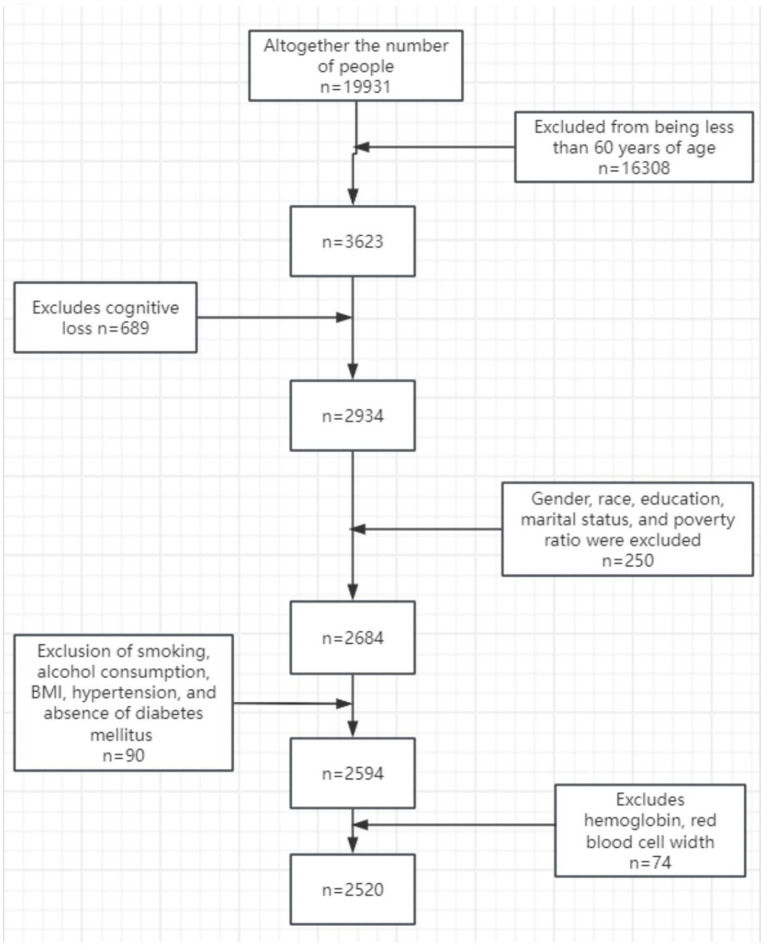
Participant selection flowchart.

For this study, data from 2 cycles of the NHANES conducted between 2011 and 2014 were acquired. The following exclusion criteria were established: incomplete cognitive function assessment, missing HB and RDW values, and missing other covariates. After screening, 2,520 participants were included in the study. The selection and inclusion process of the participants is detailed in Figure 1.

### Evaluation of cognitive function

The assessment of cognitive function encompasses three fundamental components: (1) word learning and recall modules from the Consortium to Establish a Registry for Alzheimer’s Disease (CERAD); (2) the Animal Fluency test; and (3) the Digit Symbol Substitution Test (DSST).

The CERAD Word Learning subtest (CERAD W-L) evaluates both immediate and delayed learning abilities pertaining to new verbal information, which is classified within the memory sub-domain. This assessment involves three consecutive learning trials followed by a delayed recall task. Additionally, the Animal Fluency test measures categorical verbal fluency, a vital aspect of executive function. Participants are instructed to name as many animals as possible within a 1-minute time frame, earning one point for each correctly identified animal.

The DSST, a pivotal component of the Wechsler Adult Intelligence Scale (WAIS III), assesses processing speed, sustained attention, and working memory. The cumulative score derived from these three assessments is employed to establish a comprehensive cognitive function score. Individuals falling within the lowest quartile are categorized as experiencing cognitive impairment, whereas those in the higher quartiles are classified as having normal cognitive function (Brody et al., 2019).

### HRR calculations

In this study, Complete Blood Count (CBC) measurements were obtained at the Mobile Examination Centers (MECs) of the National Health and Nutrition Examination Survey (NHANES). Detailed protocols for specimen collection and processing are delineated in the NHANES Laboratory/Medical Technologists Procedures Manual (LPM). The CBC parameters were derived using the Beckman Coulter methodology, which is well-established for the counting and sizing of blood cells. To calculate the Hemoglobin-to-Red Cell Distribution Width Ratio (HRR), Hemoglobin (HGB), measured in grams per deciliter (g/dL), was divided by the Red Cell Distribution Width (RDW), with results rounded to two decimal places for accuracy. In our analysis, HRR was treated as a continuous variable, and participants were categorized into tertiles based on their HRR values for further evaluation. In this study, HRR was identified as the primary exposure variable.

### Covariates

Covariates predominantly aim to control for confounding variables in this investigation. The covariates analyzed encompass gender (male and female), age (expressed in years), race (including Mexican American, Other Hispanic, Non-Hispanic White, Non-Hispanic Black, and Other Race), educational attainment (Under High School, High School, and Above High School), marital status (Married or Living with a Partner versus Living Alone), and the Poverty Income Ratio (PIR), categorized as <1, 1–3, and > 3. Participants who indicated having smoked more than 100 cigarettes in their lifetime were classified as smokers, regardless of their smoking status at the time of the interview. Individuals who reported consuming at least 12 alcoholic beverages annually were designated as drinkers. The Body Mass Index (BMI) is calculated by dividing an individual’s weight in kilograms (kg) by the square of their height in meters (m^2^). Based on BMI, participants were categorized into four groups: underweight (<18.5), normal weight (18.5–25), overweight (25–30), and obese (≥30). participants were classified as having hypertension if they responded “yes” to the inquiry, “Has a doctor or other health professional ever indicated that you have hypertension?” Diabetes was defined as a condition diagnosed by a healthcare professional.

### Statistical analysis

In this study, 2 cycles of data from the National Health and Nutrition Examination Survey (NHANES) were analyzed, with appropriate weights applied to ensure accuracy in accordance with the NHANES complex sampling framework. All analyses were performed using the statistical software R^2^ (The R Foundation) and Free Statistics software version 1.9.2. Continuous variables are presented as mean ± standard deviation (SD), whereas categorical variables are expressed as counts (*n*) and percentages (%). Participants were stratified into four distinct groups based on their cognitive function scores. Weighted Student’s *t*-tests were utilized for continuous variables, while weighted chi-square tests were employed for categorical variables. Multivariate logistic regression models were applied to examine the relationship between health-related resources (HRR) and cognitive dysfunction. Model 1 included no covariate adjustments; Model 2 adjusted for age, sex, race, education level, marital status, and the Poverty Income Ratio (PIR); and Model 3 further incorporated adjustments for smoking status, alcohol consumption, body mass index (BMI), hypertension, and diabetes. HRR was classified into three categories to explore the effects of different levels of HRR on cognitive function. Moreover, generalized additive models (GAM) and smooth curve fitting techniques were employed to investigate the nonlinear relationship between HRR and cognitive performance. Finally, subgroup analyses were conducted.

## Results

### Baseline characteristics of participants

Table 1 presents the baseline characteristics of the study population stratified by cognitive function tertiles. The analysis encompassed 2,520 participants, with mean ages ranging significantly from 66.199 years in the highest tertile to 71.897 years in the lowest tertile (*p* < 0.0001). A higher proportion of males was observed in the lower cognitive function groups (54.33% in tertile 1) compared to the higher tertiles (38.10% in tertile 3, *p* < 0.0001). Educational attainment demonstrated a notable inverse relationship with cognitive function, with 31.51% of tertile 1 participants having less than a high school education, contrasting with only 0.60% in tertile 4 (*p* < 0.0001). Health indicators such as hypertension and diabetes were significantly more prevalent in lower cognitive function groups (*p* < 0.0001). These findings underscore the intricate interplay between cognitive function, demographic factors, and health status within this population.

**Table 1:** Baseline characteristics of the study population by cognitive function tertiles.

Variables	level	Overall	1	2	3	4	*p*
*n*		2,520	624	616	616	664	
Age (mean ± SD)		69.437 ± 6.759	71.897 ± 6.804	70.719 ± 6.895	69.153 ± 6.417	66.199 ± 5.474	<0.0001
Sex (%)	Male	1218.00 (48.33)	339.00 (54.33)	341.00 (55.36)	285.00 (46.27)	253.00 (38.10)	<0.0001
	Female	1302.00 (51.67)	285.00 (45.67)	275.00 (44.64)	331.00 (53.73)	411.00 (61.90)	
Race (%)	Mexican American	213.00 (8.45)	78.00 (12.50)	49.00 (7.95)	47.00 (7.63)	39.00 (5.87)	<0.0001
	Other Hispanic	250.00 (9.92)	108.00 (17.31)	66.00 (10.71)	38.00 (6.17)	38.00 (5.72)	
	Non-Hispanic White	1256.00 (49.84)	206.00 (33.01)	288.00 (46.75)	327.00 (53.08)	435.00 (65.51)	
	Non-Hispanic Black	573.00 (22.74)	196.00 (31.41)	153.00 (24.84)	142.00 (23.05)	82.00 (12.35)	
	Other Race	228.00 (9.05)	36.00 (5.77)	60.00 (9.74)	62.00 (10.06)	70.00 (10.54)	
Education level (%)	Under high school	261.00 (10.37)	196.00 (31.51)	50.00 (8.12)	11.00 (1.79)	4.00 (0.60)	<0.0001
	High school	935.00 (37.13)	282.00 (45.34)	296.00 (48.05)	228.00 (37.01)	129.00 (19.43)	
	Above high school	1322.00 (52.50)	144.00 (23.15)	270.00 (43.83)	377.00 (61.20)	531.00 (79.97)	
Married (%)	Married or Living with partner	1458.00 (57.88)	300.00 (48.08)	360.00 (58.44)	369.00 (59.90)	429.00 (64.71)	<0.0001
	Living alone	1061.00 (42.12)	324.00 (51.92)	256.00 (41.56)	247.00 (40.10)	234.00 (35.29)	
BMI (%)	<18.5	35.00 (1.39)	14.00 (2.24)	7.00 (1.14)	5.00 (0.81)	9.00 (1.36)	0.1976
	18.5–25	635.00 (25.20)	160.00 (25.64)	160.00 (25.97)	155.00 (25.16)	160.00 (24.10)	
	25–30	889.00 (35.28)	217.00 (34.78)	237.00 (38.47)	204.00 (33.12)	231.00 (34.79)	
	≥30	961.00 (38.13)	233.00 (37.34)	212.00 (34.42)	252.00 (40.91)	264.00 (39.76)	
PIR (%)	<1	415.00 (16.47)	189.00 (30.29)	102.00 (16.56)	78.00 (12.66)	46.00 (6.93)	<0.0001
	1–3	1122.00 (44.52)	321.00 (51.44)	339.00 (55.03)	259.00 (42.05)	203.00 (30.57)	
	>3	983.00 (39.01)	114.00 (18.27)	175.00 (28.41)	279.00 (45.29)	415.00 (62.50)	
Smoke (%)	NO	1239.00 (49.21)	304.00 (48.80)	279.00 (45.29)	296.00 (48.13)	360.00 (54.22)	0.0131
	YES	1279.00 (50.79)	319.00 (51.20)	337.00 (54.71)	319.00 (51.87)	304.00 (45.78)	
Drink (%)	NO	782.00 (31.07)	237.00 (38.16)	201.00 (32.63)	186.00 (30.19)	158.00 (23.80)	<0.0001
	YES	1735.00 (68.93)	384.00 (61.84)	415.00 (67.37)	430.00 (69.81)	506.00 (76.20)	
Hypertension (%)	NO	935.00 (37.16)	180.00 (28.94)	222.00 (36.04)	221.00 (35.93)	312.00 (47.06)	<0.0001
	YES	1581.00 (62.84)	442.00 (71.06)	394.00 (63.96)	394.00 (64.07)	351.00 (52.94)	
Diabetes (%)	NO	1930.00 (76.62)	425.00 (68.22)	461.00 (74.84)	476.00 (77.27)	568.00 (85.54)	<0.0001
	YES	589.00 (23.38)	198.00 (31.78)	155.00 (25.16)	140.00 (22.73)	96.00 (14.46)	
HGB (mean ± SD)		13.754 ± 1.434	13.423 ± 1.554	13.802 ± 1.530	13.787 ± 1.336	13.990 ± 1.249	<0.0001
RDW (mean ± SD)		13.614 ± 1.193	13.839 ± 1.312	13.636 ± 1.149	13.670 ± 1.283	13.329 ± 0.954	<0.0001
HRR (mean ± SD)		1.019 ± 0.145	0.980 ± 0.153	1.021 ± 0.148	1.019 ± 0.143	1.056 ± 0.126	<0.0001

Mean (SD) for continuous variables, % for categorical variables. NHANES, National Health and Nutrition Examination Survey; PIR, poverty income ratio; BMI, body mass index; HGB, hemoglobin; RDW, red cell distribution width; HRR, hemoglobin-to-red cell distribution width ratio.

### Association between HRR and cognitive function

Table 2 illustrates the association between health-related resources (HRR) and cognitive function. Our results indicate that an increased HRR is linked to a diminished likelihood of cognitive impairment. In Model 1, which did not control for any covariates, HRR was associated with a markedly reduced odds of cognitive impairment, yielding an odds ratio (OR) of 0.02 (95% CI: 0.01–0.05, *p* < 0.001). This result finding indicates that higher HRR is strongly linked to improved cognitive function. Model 2 introduced adjustments for demographic variables, including age, sex, race, education level, marital status, and poverty-to-income ratio (PIR). In this model, the association remained significant, with an OR of 0.11 (95% CI: 0.03–0.46, *p* = 0.004), suggesting that even after accounting for these demographic characteristics, higher HRR continues to correlate with lower odds of cognitive impairment. Model 3 further refined the analysis by adjusting for additional health-related factors, including body mass index (BMI), smoking status, alcohol consumption, hypertension, and diabetes. The results retained statistical significance, with an OR of 0.18 (95% CI: 0.04–0.78, *p* = 0.026). This finding underscores the robustness of the association between HRR and cognitive function. Additionally, when total HRR was stratified into tertiles, this association remained statistically significant. Participants in the second tertile (Q2) exhibited significantly lower odds of cognitive impairment compared to the reference group (Q1), with an OR of 0.4 (95% CI: 0.31–0.51, *p* < 0.001) in Model 1, and an OR of 0.58 (95% CI: 0.36–0.94, *p* = 0.032) in Model 3. Similarly, those in the third tertile (Q3) demonstrated a stronger association, with ORs of 0.3 (95% CI, 0.22–0.41, *p* < 0.001) in Model 1 and 0.58 (95% CI, 0.37–0.91, *p* = 0.022) in Model 3. The trend analysis across HRR tertiles was significant for all models, with *p*-values of <0.001, 0.005, and 0.023.

**Table 2:** Association between HRR and cognitive function.

Variable	Model 1 OR (95% CI), *p*-value	Model 2 OR (95% CI), *p*-value	Model 3 OR (95% Cl), *p*-value
HRR	0.02 (0.01, 0.05) < 0.001	0.11 (0.03, 0.46) 0.004	0.18 (0.04, 0.78) 0.026
Trisections of HRR			
Q1	1.0 (Reference)	1.0 (Reference)	1.0 (Reference)
Q2	0.4 (0.31, 0.51) < 0.001	0.53 (0.34, 0.83) 0.007	0.58 (0.36, 0.94) 0.032
Q3	0.3 (0.22, 0.41) < 0.001	0.52 (0.35, 0.79) 0.004	0.58 (0.37, 0.91) 0.022
*p* for trend	<0.001	0.005	0.023

Model 1: no covariates adjusted; Model 2: adjusted for age, sex, race; education level, married and PIR; Model 3: adjusted for age, sex, race; education level, married, PIR, BMI, smoke, drink, hypertension, diabetes.

**Figure 2:**
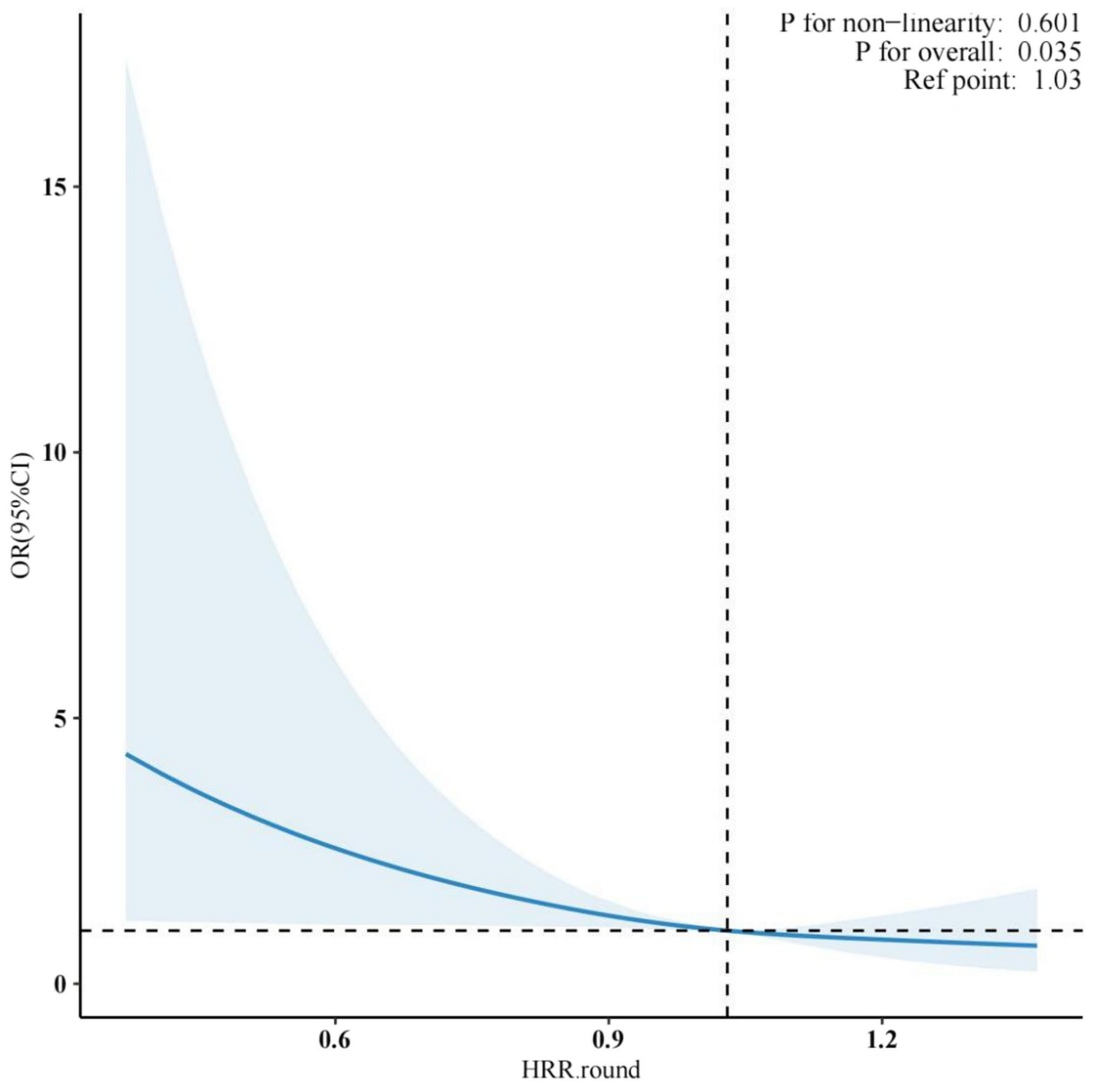
Relationship between HRR and cognitive function.

As shown in Figure 2, The dose–response relationship between hemoglobin-to-red blood cell distribution width ratio (HRR) and cognitive function was evaluated using restricted cubic spline (RCS) regression with four knots. The likelihood ratio test for nonlinearity yielded a *p*-value of 0.601, indicating no significant deviation from linearity. However, the overall model demonstrated a statistically significant association (*p* = 0.035), suggesting a linear trend between HRR and cognitive impairment risk. The reference point was set at an HRR of 1.03, where the odds ratio (OR) for cognitive dysfunction was 1.00 (95% confidence interval [CI]: referent). As HRR decreased below this threshold, a progressive increase in cognitive impairment risk was observed. Specifically, at an HRR of 0.6, the adjusted OR rose to 1.45 (95% CI: 1.12–1.88), while higher HRR values above 1.2 correlated with a nonsignificant protective trend (OR: 0.92, 95% CI: 0.79–1.08). The inflection point near the reference value (1.03) underscored its potential clinical relevance as a risk stratification marker. These findings align with the hypothesis that diminished HRR, reflecting impaired oxygen-carrying capacity and heightened inflammatory stress, may exacerbate neuronal vulnerability. The linear association emphasizes HRR’s utility as a continuous predictor rather than a threshold-dependent biomarker in cognitive health assessments.

### Subgroup analysis

To examine the impact of various factors on the relationship between cognitive function and health-related resources (HRR), we conducted a subgroup analysis. This analysis took into account variables such as sex, education level, smoking status, alcohol intake, hypertension, and diabetes. As demonstrated in Table 3, In terms of sex, there is no significant difference in the association of HRR with cognitive function between males and females. Among males, the odds ratio (OR) was 0.13 (95% CI: 0.03–0.54, *p* = 0.01), suggesting that higher HRR is linked to significantly lower odds of cognitive impairment. Regarding education level, participants with education above high school exhibited a pronounced protective effect, with an OR of 0.04 (95% CI: 0.00–0.58, *p* = 0.02). This suggests that higher educational attainment may significantly enhance the protective benefits of HRR on cognitive function. The analysis of smoking status revealed a *p*-value of 0.502 for interaction, indicating no significant difference in the association based on smoking habits. Non-smokers exhibited significantly reduced odds of cognitive impairment, with an OR of 0.09 (95% CI: 0.02–0.37, *p* = 0.002). When considering alcohol consumption, non-drinkers had a notably lower odds of cognitive impairment, with an OR of 0.02 (95% CI: 0.00–0.22, *p* = 0.004), while those who consume alcohol exhibited an OR of 0.72 (95% CI: 0.08–6.13, *p* = 0.75), indicating significant protective effect associated with no drinking. For the subgroup analysis on hypertension, the interaction test yielded a *p*-value of 0.898, suggesting no significant differences in the HRR-cognitive function relationship based on hypertension status. However, individuals without hypertension had an OR of 0.20 (95% CI: 0.01–3.40, *p* = 0.24), while those with hypertension exhibited an OR of 0.17 (95% CI: 0.03–0.98, *p* = 0.05), indicating a statistically significant protective effect. Lastly, the interaction test for diabetes yielded a p-value of 0.768, indicating no significant differential effect. Individuals without diabetes displayed an OR of 0.20 (95% CI: 0.03–1.19, *p* = 0.07), while those with diabetes had an OR of 0.16 (95% CI: 0.02–1.10, *p* = 0.06), suggesting a trend toward a protective effect. Overall, these subgroup analyses highlight the nuanced relationship between HRR and cognitive function across various demographic and health-related factors.

**Table 3:** Subgroup analysis and interaction tests of HRR and cognitive function.

Subgroup	OR (95% CI) *p* value	*p* for interaction
Sex		0.763
Male	0.13 (0.03, 0.54) 0.01	
Female	0.17 (0.02, 1.48) 0.1	
Education level		0.153
Under high school	1.49 (0.08, 28.35) 0.77	
High school	0.35 (0.10, 1.21) 0.09	
Above high school	0.04 (0.00, 0.58) 0.02	
Smoke		0.502
NO	0.09 (0.02, 0.37) 0.002	
YES	0.33 (0.03, 4.22) 0.37	
Drink		0.057
NO	0.02 (0.00, 0.22) 0.004	
YES	0.72 (0.08, 6.13) 0.75	
Hypertension		0.898
NO	0.20 (0.01, 3.40) 0.24	
YES	0.17 (0.03, 0.98) 0.05	
Diabetes		0.768
NO	0.20 (0.03, 1.19) 0.07	
YES	0.16 (0.02, 1.10) 0.06	

Adjusting for age, sex, race, education level, married, BMI, PIR, smoke, drink, hypertension and diabetes.

## Discussion

This study explored the relationship between hemoglobin to red blood cell distribution-width ratio (HRR) and cognitive function through the NHANES database. We analyzed the data from 2011 to 2014, adjusted for a number of confounders, and still found that HRR was negatively associated with cognitive function, that is, higher HRR was associated with a lower risk of cognitive dysfunction, and we also performed subgroup analyses, which showed that this result remained stable across subgroups.

This study is the first cross-sectional study of HRR and cognitive function, the ratio of hemoglobin, which carries oxygen and carries it to tissues and organs throughout the body, to the width of red blood cells, an indicator of the degree of variation in the size of red blood cells, which is important in the differential diagnosis of anemia. HRR can better reflect the function of red blood cells and is also a new inflammatory marker. At present, many studies have suggested that HRR is related to various diseases. Ning et al. (2024) showed that HRR was significantly negatively correlated with chronic kidney disease. Xie et al. (2024) found that HRR was negatively linked to the three-month outcomes in patients with acute ischemic stroke. Zeng (2024) studied the relationship between HRR and depression in the elderly. Liu et al. (2024) studied the predictive effect of HRR on mortality in patients with COPD.

Hemoglobin (Hb) and Red Blood Cell Distribution Width (RDW) are two important hematological parameters that have garnered attention in recent years due to their potential associations with cognitive function. Involving intricate mechanisms of oxygen transport, cellular metabolism, and neuroinflammation. Hemoglobin is a vital protein found in red blood cells (RBCs) that binds oxygen in the lungs and facilitates its transport to tissues, including the brain (Ahmed et al., 2020). The brain is highly sensitive to fluctuations in oxygen levels, as it relies on aerobic metabolism to produce ATP, the energy currency required for neuronal function and survival (Huang et al., 2022). When hemoglobin levels are low, it can lead to insufficient oxygen delivery to neuronal cells (Yang et al., 2022). This hypoxic environment can impair synaptic plasticity, reduce neurotransmitter synthesis, and ultimately affect cognitive processes such as learning and memory. The central nervous system (CNS) is particularly vulnerable to hypoxia due to its high metabolic demands (Tan et al., 2018), and prolonged periods of low oxygen can result in irreversible neuronal damage and cognitive decline. A reduction in hemoglobin levels can concurrently lead to decreased cerebral blood perfusion, which may induce neuroinflammation and oxidative stress-mediated production of amyloid-beta (Aβ) in the brain (Cai et al., 2017; Salminen et al., 2017). Neuroinflammatory responses can also result in iron metabolism disorders, potentially exacerbating neurodegeneration (Gao et al., 2025). Furthermore, hemoglobin reduction can cause microvascular damage, increasing the risk of cerebrovascular events and white matter hyperintensities (Borzage et al., 2016), both of which can negatively impact cognitive function. Park et al. (2016) found that decreased hemoglobin levels were associated with cortical thinning not only in frontal regions but also in the lateral temporal and medial parietal regions, which are, respectively, responsible for language and memory functions. On the other hand, RDW measures the variability in red blood cell size. The predominant factor contributing to elevated RDW in older adults is the “anemia of chronic diseases” (Beydoun et al., 2020). Elevated RDW has also been linked to systemic heightened blood viscosity (Patel et al., 2010) as evidenced by increased fibrinogen levels, diabetes mellitus, a history of coronary heart disease, and a history of cerebrovascular accidents (Tajuddin et al., 2017). In addition, Lippi et al. (2009) found that the red cell distribution width (RDW) is closely associated with circulating inflammatory markers, such as high-sensitivity C-reactive protein (hs-CRP), as well as the erythrocyte sedimentation rate (ESR). These findings suggest that RDW plays a significant role in the body’s inflammatory and oxidative stress responses, which can subsequently impact cognitive function. Inflammation and oxidative stress are known to influence brain health and cognitive performance. Chronic inflammation can lead to damage in brain tissues, affecting neural communication and synaptic plasticity (Geng et al., 2025). Oxidative stress, caused by an imbalance between free radicals and antioxidants, can also contribute to cellular damage in the brain (Haghi et al., 2025). Both of these processes can impair cognitive functions such as memory, attention, and executive function. Each of these conditions seems to be intricately intertwined with cognitive impairment, cognitive decline, dementia, or the neuropathology of dementia (Wan et al., 2020). The Hb/RDW ratio thus provides a more nuanced view of hematological health. A high Hb level coupled with a low RDW may indicate a well-functioning erythropoietic system capable of effectively delivering oxygen to the brain. In contrast, a low Hb level combined with a high RDW may suggest compromised oxygen transport and increased variability in red blood cell function, both of which can adversely impact cognitive health (Winchester et al., 2018).

Recent studies have shown that a low Hb/RDW ratio is associated with an increased risk of cognitive impairment and dementia. This may be attributed to the dual effects of low hemoglobin (leading to inadequate oxygenation of the brain) and high RDW (indicating potential underlying pathology and inflammation). Neuroinflammation, often exacerbated by chronic conditions and poor oxygen delivery, can lead to neuronal injury and accelerate cognitive decline (Du et al., 2020). Moreover, the Hb/RDW ratio may also reflect systemic processes such as oxidative stress (Padberg et al., 2020). A higher RDW may signify an influx of younger, less functional red blood cells, which are less capable of effective oxygen transport (Meng et al., 2020). This inefficiency can lead to increased oxidative stress in the brain, further damaging neuronal cells and exacerbating cognitive decline (Chen et al., 2022).

While our study highlights hypoxia and inflammation as plausible pathways linking HRR to cognitive dysfunction, we acknowledge the potential interplay between HRR and other established risk factors for cognitive decline. For instance, systemic inflammation, as reflected by elevated RDW, may synergize with metabolic disorders (e.g., diabetes or dyslipidemia) to exacerbate neuronal damage through oxidative stress and endothelial dysfunction. Chronic inflammation could amplify the production of pro-inflammatory cytokines, which are known to disrupt blood–brain barrier integrity and promote neuroinflammation, thereby accelerating cognitive impairment. Additionally, HRR’s association with anemia and microvascular insufficiency may intersect with cardiovascular risk factors (e.g., hypertension, atherosclerosis), further compromising cerebral perfusion and oxygen delivery. Lifestyle factors such as physical inactivity or poor dietary patterns (e.g., iron or vitamin B12 deficiency) could also modulate HRR levels, creating a bidirectional relationship where suboptimal nutrition exacerbates hematological dysfunction, while impaired oxygen transport perpetuates metabolic inefficiencies in the brain. Future mechanistic studies should explore how HRR interacts with these multifactorial pathways, particularly in longitudinal cohorts with detailed biomarker profiling.

The advantages of this study are as follows: First, this study is based on NHANES data, with a large and representative sample size, and we have carried out weight analysis; Second, we adjusted for multiple covariates to make the results more robust. In addition, we also conducted subgroup analysis, but our study still has many limitations. First, the study is a cross-sectional study, and we cannot determine causality. Second, although our study accounted for a multitude of confounding factors, the possibility of residual bias arising from unmeasured variables (such as diet, physical activity levels, or subclinical inflammation) cannot be entirely excluded. Finally, the study is based on specific countries and ethnicities and needs to be further explored in other countries and regions.

## Conclusion

HRR serves as a valuable metric in evaluating cognitive function among the elderly population. A diminished HRR is indicative of a heightened risk of cognitive decline.

## Data Availability

The raw data supporting the conclusions of this article will be made available by the authors, without undue reservation.

## Footnotes

^1^ https://wwwn.cdc.gov/Nchs/Nhanes/

^2^ http://www.R-project.org
